# Rapid effects of valproic acid on the fetal brain transcriptome: Implications for brain development and autism

**DOI:** 10.21203/rs.3.rs-3684653/v1

**Published:** 2024-01-10

**Authors:** Susan G. Dorsey, Evelina Mocci, Malcolm V. Lane, Bruce K. Krueger

**Affiliations:** 1 Department of Pain and Translational Symptom Sciences, University of Maryland School of Nursing, Baltimore, MD 21201; 2 Institute for Genome Sciences, University of Maryland School of Medicine, Baltimore, MD 21201; 3 Translational Toxicology/Department of Epidemiology and Public Health, University of Maryland School of Medicine, Baltimore, MD 21201; 4 Departments of Physiology and Psychiatry, University of Maryland School of Medicine, Baltimore, MD 21201

## Abstract

There is an increased incidence of autism among the children of women who take the anti-epileptic, mood-stabilizing drug, valproic acid (VPA) during pregnancy; moreover, exposure to VPA *in utero* causes autistic-like symptoms in rodents and non-human primates. Analysis of RNA-seq data obtained from E12.5 fetal mouse brains 3 hours after VPA administration to the pregnant dam revealed that VPA rapidly and significantly increased or decreased the expression of approximately 7,300 genes. No significant sex differences in VPA-induced gene expression were observed. Expression of 399 autism risk genes was significantly altered by VPA as was expression of 255 genes that have been reported to play fundamental roles in fetal brain development but are not otherwise linked to autism. Expression of genes associated with intracellular signaling pathways, neurogenesis, and excitation-inhibition balance as well as synaptogenesis, neuronal fate determination, axon and dendritic development, neuroinflammation, circadian rhythms, and epigenetic modulation of gene expression was dysregulated by VPA. The goal of this study was to identify mouse genes that are: (a) significantly up- or down-regulated by VPA in the fetal brain and (b) known to be associated with autism and/or to play a role in embryonic neurodevelopmental processes, perturbation of which has the potential to alter brain connectivity and, consequently behavior, in the adult. The set of genes meeting these criteria provides potential targets for future hypothesis-driven studies to elucidate the proximal causes of errors in brain connectivity underlying neurodevelopmental disorders such as autism.

## INTRODUCTION:

Autism is a neurodevelopmental disorder (NDD) characterized by social interaction deficits including language, and repetitive, stereotyped behavior with restricted interests ^1^. Autistic individuals may also display an increased incidence of intellectual disability (ID), anxiety and seizures. The reported incidence of autism has increased dramatically over the last two decades ^2^, with some estimates now as high as 1:44 ^3^. The severity of autism varies widely, ranging from high-functioning with minimal disability, to severely afflicted, where aggressive, self-destructive repetitive behaviors pose a threat to the safety of the patient, caregivers, and family. The term “autism spectrum disorder” (ASD) reflects this symptomatic variability. The neurological mechanisms underlying ASDs are not understood nor is it known whether autism is a single disorder or multiple disorders sharing common core features. The diagnosis of autism typically is made at around two years of age when the child fails to meet normal milestones for social development and language. However, it has become increasingly evident that autism arises before birth: infants destined for an autism diagnosis fail to show normal attention to faces ^4^. Epidemiological studies also indicate that the onset of autism occurs during fetal development; one such study found that folate supplementation for pregnant women reduced the incidence of autism, but only when administered between two weeks before and four weeks after conception ^5^.

Twin studies have revealed that 60 to 88% of autism cases are inherited ^6,7^, however, many cases have been linked to *in utero* exposure to environmental factors such as pharmaceuticals, air pollution, insecticides, and maternal infection ^8,9^. For example, exposure to the anti-epileptic, mood stabilizing drug, valproic acid (VPA; Depakote^®^) ^10^ or the organophosphate insecticide, chlorpyrifos ^11^, increases the incidence of autism and other NDDs in the offspring of women who are exposed during pregnancy. Another environmental cause of autism is maternal immune activation (MIA) in which pregnant women who have systemic bacterial or viral infections with a hyper-immune response have an increased incidence of autism in their children ^12–14^.

Studying the etiology of autism in humans is limited to epidemiological approaches implicating genetic variants (single nucleotide polymorphisms, copy number variants, and single-gene syndromic mutations). The Simons Foundation Autism Research Initiative (SFARI) has compiled a list of 1,115 human genes (https://gene.sfari.org/database/human-gene/), referred to as the “SFARI List” below, for which there is evidence for association with autism based on genome-wide association studies (GWAS).

Hypothesis-driven experiments to determine cause and effect need to be done in animal models, which display a range of behaviors that are remarkably similar to the core symptoms of autism ^15–17^. These autistic-like behaviors have been reported in transgenic animals lacking ASD-linked genes such as *Cntnap2* and *Shank2* ^18,19^, supporting the conclusion that pathogenic mutations in these genes are causative for syndromic autism and leading to the widespread use of these transgenic mice as animal models ^20,21^. Systemic administration of VPA, chlorpyrifos or induction of MIA in genetically normal pregnant rodents also leads to increased autistic-like behaviors in their offspring ^12,14,16,17^. Unlike transgenic mouse models, these environmental toxicity models of autism provide the opportunity to control the timing of exposure of the fetus to the toxic stressor, as will be discussed below. Gene expression analyses of the brains of mice in the VPA and MIA models have been conducted using RNA microarray analysis or next-generation RNA sequencing (RNA-seq). In most of those animal studies, the fetus was exposed to the drug during mid-gestation, but RNA expression was analyzed in postnatal or adult animals (e.g., see refs 22–26).

There are two competing, but not mutually exclusive, theories about the role of differential gene expression in autism. In one, abnormal gene expression in the child or adult at the time of behavioral testing, is responsible for autistic-like behavior. The second posits that abnormal gene expression, due to genomic variants or *in utero* environmental stressors, interferes with one or more critical steps in the “program” controlling fetal brain development. Such errors could carry forward throughout life leading to a brain with subtle anatomical or connectivity defects that underlie the abnormal development of an autistic brain. This has been referred to as a “presymptomatic signature” ^27^. The present study focused on the second hypothesis by examining altered gene expression in fetal brains when the environmental stressor (VPA) is still present; these genes need not be continuously dysregulated throughout life. Consequently, genes associated with critical steps in early brain development such as neurogenesis, neuron fate specification, axon and dendrite growth, and synaptogenesis are of particular interest.

The reported incidence of autism is about 4-times higher in males than in females ^28^. Sex differences in neural function are generally considered to be mediated by sex hormones acting from late prenatal brain development through to the adult. Since the time of VPA administration in the present study (E12.5) is just prior to the maturation of gonads and production of sex hormones ^29^, any sex differences in gene expression observed likely would be due to sexually-dimorphic gene expression rather than to hormonal effects.

The complexity and variability of the behavioral symptoms of autism together with identification of over 1,100 ASD-associated gene variants (SFARI List) make a mechanistic understanding of the causes of the disorder a daunting task. One approach to this problem is to compare the results of multiple studies using GWAS results from human patients and gene expression results from animal models of autism, looking for common differentially-expressed genes. The subset of genes in common from both types of studies can provide a short(er) list of candidate genes that could be subjected to future hypothesis-driven studies to establish causal relationships between gene dysregulation and ASD symptoms. Meng et al. ^30^ have taken an analogous approach, identifying genes that are dysregulated by long-term VPA treatment of human forebrain organoids *in vitro* and that are also linked to autism.

The goal of the present study was to address this question using the VPA mouse model in which pregnant mice receive a single i.p. injection of VPA at gestational day 12.5 (E12.5). Fetal brains were processed for RNA-seq three hours after VPA administration; male and female fetuses were analyzed separately. Pharmacokinetic studies have demonstrated that injected VPA dissipates within 3–5 hours due to metabolism of the drug by the maternal liver ^31,32^; VPA levels in the fetal brain follow a similar trajectory ^33,34^. Consequently, with this protocol, the fetal brain receives a brief, transient exposure to VPA at a critical time for early brain development. Thus, VPA-induced molecular events occurring on E12.5 are both necessary and sufficient for the autistic-like behaviors in VPA-treated animals assessed 5–6 weeks later. Analysis of these data revealed that VPA induced a significant increase or decrease in the expression of approximately 7,300 genes, of which 399 are among the 1,115 genes on the SFARI List and at least 255 additional genes are known to play fundamental roles in the development and function of the nervous system.

## MATERIALS AND METHODS:

Timed-pregnant C57BL6 mice were generated by the University of Maryland School of Medicine Division of Veterinary Resources. All animal procedures were approved by the University of Maryland Baltimore, Institutional Animal Care and Use Committee. VPA was obtained from Sigma.

One male was paired overnight with two females; the day of separation was designated E0.5. On E12.5 pregnant females received i.p injections of VPA (400 mg/kg) in sterile saline or saline alone. 7 pregnant females received VPA and 7 received saline. Three hours after VPA administration, pregnant females were euthanized by cervical dislocation and decapitation. Fetuses were transferred to ice-cold saline, decapitated and the entire brain was removed to Trizol and disrupted for 60 sec with a Bead Beater using 0.2 mm beads. The 105 individual fetal brain samples were frozen on dry ice and stored at −80°C prior to RNA extraction and quality control (RIN = 10 for all samples).

Sex was determined by analyzing *Sry* and *Gapdh* RNA derived from the torso of each fetus by PCR. Males were identified by the presence of both *Sry* and *Gapdh*; *Gapdh* but not *Sry* RNA was expressed in females ^35^. One male and one female brain from each of the 7 pregnancies at each condition were processed for RNA-seq by the University of Maryland Institute for Genome Sciences.

Libraries were prepared from 25 ng of RNA using the NEB Ultra II Directional RNA kit. Samples were sequenced on an Illumina NovaSeq 6000 with a 150 bp paired-end read configuration. The quality of sequences was evaluated by using FastQC ^36^. The alignment was performed using HiSat (version HISAT2–2.0.4) ^37^ and *Mus musculus* GRCm38 as reference genome and annotation (version 102). Aligned bam files were used to determine number of reads by gene using HTSeq ^38^. On average, we sequenced 187,000,000 reads. 96.6% of them properly mapped the reference: 87% mapped exons, 2.4% mapped introns and the remaining 10.6% mapped intergenic regions.

Differential gene expression between mice treated with VPA and saline controls was conducted using DESeq2 ^39^, which models gene counts using the negative binomial distribution. P-values were corrected for false discovery rate (FDR) using the Benjamini-Hochberg procedure; p_FDR_ ≤ 0.025 and log_2_-fold change ≥0.32 was used as the criterion for significance. Differentially expressed genes due to VPA treatment were tested for enrichment in SFARI autism risk genes (https://gene.sfari.org/database/human-gene/) using Fisher’s Exact Test. Significance of sex-differences in FC was analyzed by 2-way ANOVA using the Limma-Voom tool ^40^.

## RESULTS:

### RNA-seq.

19,721 individual genes were analyzed in fetal mouse brain by RNA-seq. Male and female brains were analyzed separately. The raw data showing base counts (mean of male and female counts) for each gene in each of seven independent fetal brain samples of each sex, each from a different pregnancy, are shown in Supplemental Table S1, i.e., n = 7 fetal brains from 7 different pregnancies for each sex/condition. Only genes with base counts ≥100 were analyzed further. Genes that increased or decreased in response to VPA by < 1.25-fold and > −1.25-fold and those with adjusted p-values (p_FDR_) > 0.025 were also filtered out; a gene was included if VPA increased or decreased its expression by ≤ 1.25-fold and ≥ −1.25-fold in only one sex. Shown in Supplemental Table S2 are the 6516 genes significantly affected by VPA (p_FDR_ ≤ 0.025) in both sexes, 498 in females only and 280 in males only (listed in three separate sections in Table S2). These apparent sex differences were due to variability in the replicates of one of the sexes (i.e., p_FDR_ > 0.025).

Throughout this report, a positive fold-change (FC) indicates that VPA increased gene expression; a negative FC indicates that VPA decreased expression such that the FC is the ratio of control to VPA gene expression. For example, FC = −3.0 indicates that V/C = 0.33, a 67% reduction by VPA.

### Curating the mouse genes dysregulated by VPA in the fetal brain.

The 7294 genes that were significantly altered by VPA (Table S2) were further analyzed in two ways. First, they were merged with the genes on the SFARI List identifying 399 common genes (Table S3). It should be noted that inclusion on the SFARI List is based on GWAS; in many cases, a mechanistic role in neither brain development nor the etiology of autism is known.

Second, we identified 255 genes (Table S4) significantly altered by VPA, not on the list of SFARI risk genes but having a documented role in brain development including intracellular signaling, neurogenesis, and excitation-inhibition balance as well as 15 additional categories (c.f., Table 3 and Supplementary Discussion). As will be discussed below, although these 255 genes have not been associated with autism in GWAS, it is plausible that changes (either positive or negative) in the expression of their gene products in the fetal brain might adversely affect the trajectory of brain development leaving a permanent “signature” ^27^ on brain structure and connectivity leading to autistic-like behavior in the adult.

### No significant sexually-dimorphic effects of VPA.

**Figure 1.**
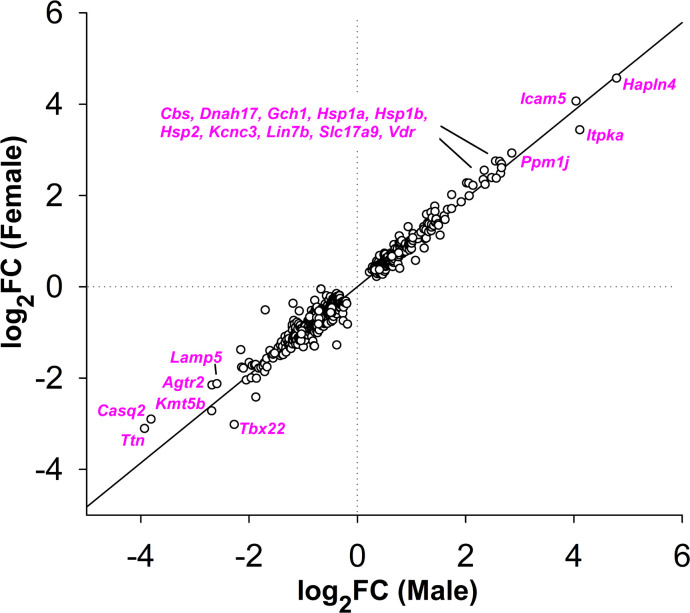
Log_2_ fold-change (FC) induced by fetal VPA exposure plotted as females versus males for the 654 genes in Tables S3 and S4. Deviations from the regression line (r^2^=0.973) indicate possible sex differences. The gap in the data reflects the ±1.25-fold FC cut-off. 20 genes (magenta) were upregulated by > 5-fold or downregulated > 80% by VPA (average of males and females).

Figure 1 is a plot of the log_2_ fold-change (FC) of males versus females for the 654 genes in Tables S3 and S4. Deviations from the regression line indicate possible sex differences; however, in no case was the effect of VPA found to be significantly different between males and females. 20 genes (magenta) were upregulated by > 5-fold or downregulated > 80% by VPA (average of males and females).

### Limitations:

1. The results reported here are restricted to VPA-induced changes in RNA levels; the extent to which these changes reflect commensurate changes in the expression levels of the proteins encoded by these genes is not known. Although in many cases, RNA and protein levels are correlated, the extent to which RNA levels of a given gene track its corresponding protein levels may vary.
2. These results represent VPA-induced changes in gene expression at a time single point in time (3 hr after VPA administration on E12.5). Whether the changes are transient and reverse as the VPA levels dissipate, as has been reported for *Bdnf* ^41,^ or are persistent, is not known. It is also not known whether the same VPA induced changes would be observed after VPA administration on a different gestational day.
3. There are many genes for which expression is increased or decreased by ≥ 1.25-fold or ≤ −1.25-fold in response to VPA but the FDR-corrected p-value did not reach the ≤ 0.025 criterion applied in this study. Higher statistical power would be needed to determine whether these changes are significant.
4. The ± 1.25 FC cutoff used here is arbitrary; it is possible that smaller changes in the expression of one or more critical genes have profound effects on fetal brain development and these would not be identified in this analysis.
5. Only a fraction of the approximately 7,300 genes whose expression is significantly affected by VPA were curated in this study. It is likely that other genes, in addition to those shown in Tables S3 and S4, could play a role in mediating the effects of VPA on fetal brain development.

### Reproducibility:

The stimulation by VPA of *Bdnf* expression (Table S4) has been independently confirmed by quantitative RT-PCR ^35,41^.

By separately analyzing gene expression in male and female brains, the results of this study were effectively replicated with independent biological samples (7 of each sex ± VPA, each from a different pregnancy). With very few exceptions, male and female gene expression levels were similar (c.f., Figure 1), demonstrating reproducibility across independent samples.

## DISCUSSION:

The overarching hypothesis for this research is that NDDs involving ID such as ASDs are the result of abnormal connections within and among multiple brain regions. Normal connectivity is established beginning during fetal brain development as the various brain regions become populated with specific classes of neurons that subsequently connect with other neurons to establish the complex neuronal networks that underlie cognition and behavior. Although these networks are refined during late prenatal and postnatal brain development, it is likely that certain early prenatal developmental epochs are particularly vulnerable to alterations in the neurodevelopmental “program”. Although it is by no means clear that the fetal rodent brain accurately recapitulates the autistic human brain during prenatal development, the VPA model enables experimental control of the timing of exposure to an environmental factor that causes abnormal behavior resembling autism. In the present experimental paradigm, VPA is administered to the pregnant dam at E12.5, a time when multiple early neurodevelopmental events critical for brain organization and connectivity are occurring. These include proliferation of neural progenitors (NPs), determination of the neuronal fate of these NPs, migration of newborn neurons away from the proliferative ventricular zones, differentiation of NPs to establish a mature neuronal phenotype, extension and branching of axons and dendrites and establishment of synaptic connections. The goal of this study was to identify genes that are:

- rapidly and significantly up- or down-regulated by VPA in the fetal mouse brain (Table S2) *AND*
- known to be linked to autism in GWAS (Table S3) or to play a role in embryonic neurodevelopmental processes, perturbation of which has the potential to alter brain connectivity in the postnatal and adult brain (Table S4).

The set of genes meeting these criteria would provide potential targets for future hypothesis-driven approaches to understanding the underlying proximal causes of defective brain connectivity in NDDs such as autism.

It was not practical to vet each of the nearly 7,300 VPA-dysregulated genes for a potential role in abnormal brain development. However, as described below and in the Supplemental Discussion, the 255 genes in Table S4 and some of those in Table S3 have been reported to play substantial roles in specific aspects of fetal brain development. Consequently, it is plausible that the up- or downregulation of one or more of those genes could contribute to the abnormal connectivity in subjects with NDDs. The validity of this potential contribution could be tested in animals by determining the effect of manipulating the expression of individual (or combinations of) genes in the fetal brain on subsequent behavior.

### A common subset of genes in GWAS and VPA-fetal mouse brain studies.

Several GWAS have identified genes linked to autism (see SFARI List). Here we tabulated the findings of four GWAS studies ^42–45^ and one report categorizing autism risk genes potentially involved in neurogenesis ^46^. As shown in Table 1, many of the genes identified in these reports are also dysregulated by VPA in the fetal mouse brain (Tables S3 and S4). Genes discussed in the respective papers are listed in each column in Table 1; genes that are also dysregulated by VPA in the present study are shown in **bold**. Five VPA-regulated genes in Tables S3 and S4 that also appear in all five of the published reports are in 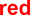. (*Arid1b*, *Dyrk1a*, *Pogz*, *Pten*, *Tbr1*). Six genes in Tables S3 and S4 that also appear in four of the five other studies are in 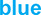. (*Adnp*, *Ash1l*, *Chd2*, *Kmt5b*, *Tcf7l2*, *Wac*). There were four genes (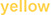) identified in all five previously published studies that were not affected by VPA in the fetal mouse brain. *Chd2*, which is listed in three of the four published studies, and *Chd3*, (50% reduced by VPA), are both SFARI genes and have been reported to have similar functions to *Chd8*. Of the 11 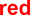 and 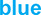 genes in Table 1, three (*Adnp*, *Dyrk1a*, *Pten*) appear in Table 2 as VPA-regulated, autism-associated genes involved with the structural stability of neurons ^47^ (see below).

### VPA dysregulation of high-confidence (hc)ASD genes in mid-fetal layer 5/6 cortical projection neurons.

Willsey et al. ^48^ identified 9 hcASD genes that are expressed in layer 5/6 projection neurons in the fetal human brain. Three of these genes (*Dyrk1a*, *Pogz*, *Tbr1*) were downregulated by VPA in this study (Table S3). The authors used the nine hcASD genes as seed genes for co-expression network analysis, which revealed 10 probable ASD genes, of which two (*Bcl11a*, *Nfia*) were down-regulated and one (*Aph1a*) was up-regulated by VPA (Table S4). All of these genes are on the SFARI List (Table S3) and the three seed genes were identified in all 5 reports analyzed in this study (Table 1). These findings raise the possibility that dysregulation of one or more of these genes in developing layer 5/6 projection neurons in the cortical plate of the fetal brain contributes to permanent connectivity defects underlying autistic-like behaviors.

### Autism-associated genes and structural stability of neurons.

Lin et al. ^47^ tabulated genes linked to autism in GWAS that have also been reported to be involved in the structural stability of neurons. These genes were further sorted into three categories, viz., “neurite outgrowth”, “spine/synapse formation” and “synaptic plasticity”. 61 genes were assigned to these three categories although some genes appeared in two or all three categories, resulting in 29 different genes among the three categories (Table 2). Shown in red 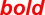 are 10 of these genes (35%) that were significantly up- or downregulated by VPA (i.e., in Table S3). Dysregulation of one or more of these genes (due to gene variants or fetal exposure to VPA) could alter the structure and function of synapses throughout brain development.

Examination of Tables 1 and 2 revealed that *Pten* and *Dyrk1a* dysregulated by VPA and are common to both the Lin et al. study ^48^ and to all five of the GWAS. Consequently, these two genes may be good candidates for investigating the molecular basis for the proximal causes of autism.

### Biological roles of genes dysregulated by VPA.

Table 3 shows the biological function of 189 different genes that were significantly dysregulated by VPA in the E12.5 fetal brain. One or more developmental errors caused by these changes could induce a “signature” of circuit defects in the fetal brain, resulting in abnormal behavior in juveniles and adults. Many of the genes affected by VPA are involved in intracellular signaling pathways, which could mediate a wide variety of developmental processes. Some examples of these processes are discussed in the following sections (“Intracellular Signaling”, “Neurogenesis”, “Excitation-Inhibition”). The rationale for inclusion of the genes in the remaining 15 categories together with supporting references is provided in Supplemental Discussion. In this discussion, the change in gene expression induced by VPA in the fetal brain at E12.5 (expressed as fold-change) is shown in parentheses (mean of males and females).

#### Intracellular signaling.

**Figure 2.**
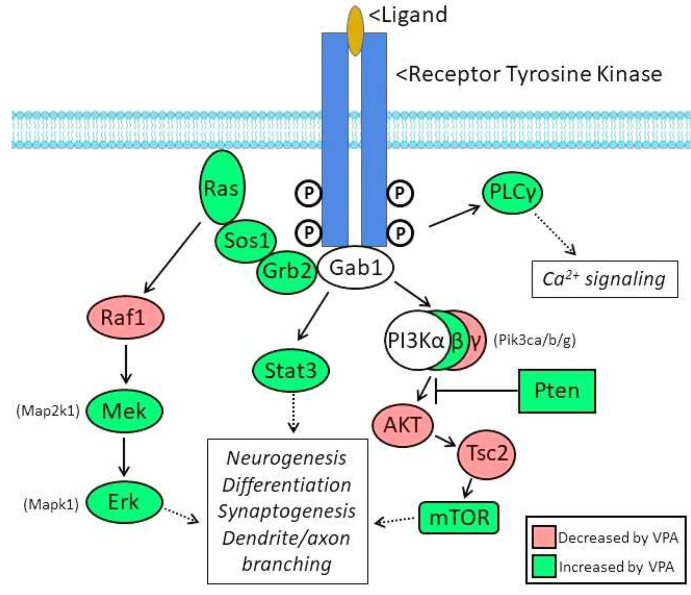
Schematic diagram of multiple signaling pathways downstream from receptor tyrosine kinases (RTKs), which mediate the action of ligands active in the developing brain. The two subunits of the RTKs are trans-autophosphorylated upon activation by extracellular ligand with the phosphorylated tyrosines serving as docking sites which initiate downstream signaling. Components in red are significantly downregulated by VPA in the fetal brain; those in green are upregulated by VPA.

As summarized in Figure 2, the genes encoding at least 10 members of the canonical signaling pathways downstream from receptor tyrosine kinase receptors are significantly up-regulated by VPA in the fetal brain (green); four are down-regulated (red). ***Pten* (+1.4)** (Phosphatase and tensin homolog), a negative regulator of the PI3K-AKT-mTor signaling pathway, has a particularly strong association with autism, appearing in all GWAS (Table 1) and associated with the structural stability of neurons (Table 2) ^47^.

Twelve additional genes associated with intracellular signaling are included in Table 3. Thus, abnormal signaling via these pathways is a potential mechanism by which VPA could interfere with the establishment of normal brain connectivity. Meta-analysis of GWAS and copy number variant studies of autism-related genes revealed that three signaling networks, regulating steroidogenesis, neurite outgrowth and excitatory synaptic function, were enriched ^48^. A-kinase anchoring proteins (AKAPs) functionally integrate signaling cascades within and among these networks ^49^; VPA decreased and increased ***Akap8* (−1.3)** and ***Akap8l* (+1.6)** expression in fetal mouse brains.

***Dyrk1b* (−2.2)** (which encodes dual-specificity tyrosine phosphorylation regulated kinase 1B) is a mediator of double-stranded DNA break repair ^50^ and regulates hedgehog signaling by activating the mTor/AKT pathway ^51,52^. *Dyrk1b* has been linked to metabolic syndrome and autism ^53^. The 55% reduction in *Dyrk1b* expression induced by VPA could contribute to autistic-like behavior by dysregulating hedgehog or mTor/AKT signaling. ***Ppp1r1b* (+3.7)**, encodes the dopamine- and cAMP-regulated neuronal phosphoprotein (DARPP-32). DARPP-32 amplifies and/or mediates many actions of cyclic AMP-dependent protein kinase at the plasma membrane and in the cytoplasm, with a broad spectrum of potential targets and functions ^54^.

#### Neurogenesis.

Excitatory neurons in the mammalian cortex are generated from proliferating radial glia cells (RGs), which undergo mitosis at the ventricular surface to expand the pool of neuroblasts and consequently determine the final number of neurons ^55^. Neurogenesis proceeds according to a “program” that is tightly-regulated by intercellular signals from a variety of brain cells as well as the meninges. Disturbances in this regulation, for example by environmental factors such as VPA or gene mutations, have the potential to alter the neuronal population and configuration of the developing brain leading to permanent defects in connectivity that could lead to altered behavior. An example of this disruption is the effect of VPA exposure at E12.5 on the number of neocortical neurons born on E14.5 measured one day before birth (E18.5) ^56^. Thus, gene expression changes on E12.5 alter neurogenesis at E14.5, long after the VPA has dissipated.

Retinoic acid (RA) plays a number of roles in the regulation of cortical neurogenesis ^57,58^. The RA is produced in the dorsal forebrain meninges and acts at receptors on the end-feet of RGs ^59^. The latter study used a transgenic mouse with reduced expression of ***Foxc1* (−1.9)** leading to defects in forebrain meningeal formation and loss of neuron progenitor production due to failure of proliferating RGs to exit the cell cycle. This would be predicted to lead to a delay in the generation of postmitotic neurons and consequently, an expansion of the proliferating RG pool neuroprogenitor pool.

Downregulation of ***Nr2f1* (−1.8)** and ***Sfrp2* (−1.8)** would be predicted to influence the balance between proliferation and neurogenesis ^60^. Multiple studies have implicated ***Eomes* (*Tbr2*) (−1.8)** in the regulation of cortical neurogenesis ^61–66^, while ***Neurog1* (−2.2)** has been reported to play a role as a negative regulator of cortical neurogenesis ^67^.

The conversion from symmetrical to asymmetrical mitoses associated with the generation of post-mitotic neurons is regulated by the ***Plk1* (−1.7)** – ***Lrrk1* (−2.9)** – ***Cdk5rap2* (−1.3)** cascade ^68^. Sulliman-Lavie et al. ^69^ reported that ***Pogz* (−2.2)** is a negative regulator of transcription and *Pogz* deficiency upregulates expression of genes associated with ASD resulting in disruption of embryonic neurogenesis; *Pogz* was found to be associated with autism in all five GWAS (Table 1). Several members of the heterogeneous nuclear ribonucleoprotein family have been linked to NDDs including ***Hnrnph1* (−1.6), *Hnrnpk* (−1.3), *Hnrnpu* (−1.6);** expression of these genes in radial glia has been reported to be critical during neurodevelopment ^70^.

Comparison of the mechanisms controlling neurogenesis in rodents and humans has revealed that gyrification of the human brain is driven by changes in the expression of regulatory genes and growth factors ^71^. Stahl et al. ^72^ reported that, over the period E12 to E16, decreased expression of the DNA-associated protein, ***Trnp1* (+1.5)**, leads to radial expansion of the cortex and the appearance of gyri-like folding of the mouse brain. In the present study, VPA induced a 50% increase in the expression of *Trnp1* in the E12.5 fetal mouse brain; this would be predicted to inhibit or delay the shift to radial growth of the cortex. Fibroblast growth factor 2 (FGF2) has also been reported to induce gyrification in the mouse brain, having an effect opposite to that of *Trnp1* ^73^. In the present study, VPA caused a 65% reduction on the expression of ***Fgf2* (−2.9)** (Table S4). This would also be predicted to bias cortical neurogenesis toward tangential and away from radial growth which could contribute to gyrification. These findings together with the present results lead to a hypothesis that increased *Trnp1* and decreased *Fgf2* expression, induced by VPA in the fetal brain at E12.5, cause a delayed shift in radial growth resulting in autism-like behavioral defects. This hypothesis could be tested by experimentally decreasing *Trnp1* and increasing *Fgf2* expression in the mouse fetal brain exposed to VPA; these maneuvers would be predicted to normalize behavior.

WNT/β-catenin signaling plays several roles in regulating neurogenesis in the developing neocortex ^74^. ***Wnt3a* (−3.8)** regulates the timing of differentiation of neocortical intermediate progenitors into neurons ^75^. The 74% reduction in *Wnt3a* expression induced by VPA has the potential to disrupt the normal timing of cortical neurogenesis in the fetal brain. Zinc finger and BTB domain-containing 16 [***Zbtb16* (−1.8)**] has been reported to regulate cortical neurogenesis and *Zbtb16* knockout mice display a thinning of neocortical layer 6 and a reduction of TBR1-expressing neurons as well as increased dendritic spines and microglia ^76^. The VPA-induced reduction in *Wnt3a* or *Zbtb16* expression or both at E12.5 has the potential to alter the developmental program, resulting in altered cortical connectivity and, ultimately behavioral deficits.

Cortical size is regulated, in part, by BAF-170, [encoded by ***Smarcc2* (−1.6)**], a subunit of the chromatin remodeling complex, mSWI/SNF. Deletion of *Smarcc2* in mouse increases the pool of intermediate progenitors resulting in an enlarged cortex ^77^; *de novo* variants in *Smarcc2* cause a syndrome with developmental delay and ID ^78^. Mutation of a different BAF subunit, ***Arid1b* (−2.2)**, alters the production of neuronal precursor cells in human cerebral organoids ^60^; mutation of 36 high-risk ASD genes altered the fate of dorsal intermediate progenitors, ventral progenitors, and upper-layer excitatory neurons. Expression of 18 of those genes (*Adnp*, *Arid1b*, *Ash1l*, *Asxl3*, *Baz2b*, *Bcl11a*, *Chd2*, *Ddx3x*, *Irf2bpl*, *Kat2b*, *Kdm6b*, *Kmt5b*, *Mecp2*, *Pogz*, *Srcap*, *Tbl1xr1*, *Tbr1* and *Wac*) was dysregulated by VPA at E12.5 (c.f., Tables S3 and S4).

#### Excitation-Inhibition.

A widely discussed hypothesis is that autism is caused by an imbalance between neuronal excitation and inhibition due to increased excitation, reduced inhibition, or both ^60,79,80^. Consequently, alterations in the developmental program in the fetal brain that result in too many excitatory (glutamatergic) or too few inhibitory (GABAergic) neurons (or synapses) could contribute to autistic behavior. ***Camk2a* (+1.9)**, which is associated with excitatory synapses, is upregulated by 90% in response to VPA; *CAMK2A* is upregulated in the ASD superior temporal gyrus ^81^. Two related genes, ***Maf* (−1.6)** and ***Mafb* (−1.6)**, which are both downregulated by about 40% by VPA, have been reported to play redundant roles in the generation of interneurons from the fetal median ganglionic eminence; their deletion results in decreased numbers of cortical somatostatin-releasing, inhibitory interneurons ^82^, ***Gad1* (−2.5)** and ***Gad2* (−3.3)** encode isoforms of the enzyme that synthesizes the inhibitory transmitter, GABA. VPA reduced *Gad1* and *Gad2* levels by 60% and 70%, respectively; *GAD1* and *GAD2* are downregulated in the superior temporal gyrus of ASD patients ^81^. ***Insyn1* (−1.4)** encodes a component of the dystroglycan complex at inhibitory synapses; loss of *Insyn1* alters the composition of the GABAergic synapses, excitatory/inhibitory balance, and cognitive behavior ^83,84^. Using cortical assembloids together with CRISPR screening to analyze interneuron generation and migration, Meng et al. ^85^ found that ***Csde1* (−2.0)** is required for interneuron generation. Taken together, these findings are consistent with VPA increasing net brain excitation by decreasing the number of inhibitory interneurons generated during fetal brain development. These reductions in gene expression may contribute to a decrease in inhibition in mice exposed to VPA *in utero* ^86^. ***Nf1* (−1.3)** encodes NF1, which is mutated in neurofibromatosis type 1, an inherited neurocutaneous disorder associated with NDDs including autism. *Nf1* deletion results in the specific loss of parvalbumin-expressing inhibitory cortical interneurons ^87^. In the present study, *Nf1* was downregulated by about 25% in fetal mouse brains exposed to VPA. Semaphorin-4a [***Sema4a* (−1.6)**] and Semaphorin-4d [***Sema4d* (+1.9)**]**,** promote inhibitory synapse development via the postsynaptic Plexin-B1 receptor encoded by ***Plxnb1* (−1.8)** ^88^.

***Hapln1* (−3.7)** and ***Hapln4* (+25.7)**, encode extracellular matrix proteins that are components of perineuronal nets (PNNs) ^89,90^. Ramsaran et al. ^91^ reported that *Hapln1* mediates the functional maturation of hippocampal parvalbumin interneurons through assembly of PNNs; this mechanism mediates the development of memory precision during early childhood. *Hapln4* is a selective regulator of the formation of inhibitory GABAergic synapses between Purkinje and deep cerebellar nuclei neurons ^92^; the cerebellum is one of many brain regions implicated in the etiology of autism ^93^. The 70% decrease in *Hapln1* expression and the massive, 25-fold increase in *Hapln4* induced by VPA would be expected to alter PNN density, which could disrupt the connectivity of neuronal circuitry leading to cognitive behavioral deficits.

## SUMMARY:

The approach for this study can be described as “hypothesis generating” in that we began without any preconceived idea (hypothesis) as to the mechanism by which a brief, transient dose of VPA, administered to the pregnant mouse at E12.5, can cause abnormal, autistic-like behavior in her offspring many weeks later. The analysis identified approximately 7,300 genes, expression of which was significantly affected by VPA. We then identified those genes significantly affected by VPA that were a) also linked to autism by GWAS (SFARI List; Table S3) or b) not on the SFARI List (Table S4) but known to play a role in critical steps of early brain development. Interference with one or more of these steps in the fetal brain has the potential to interfere with the “program” directing brain development to create a persistent pathological “signature” that leads to abnormal neuronal circuitry in the adult, long after the increase in VPA and the initial changes in gene expression have dissipated. Of these 654 genes, at least half have known mechanisms of action associated with brain development or function, of which 189 (Table 3) are discussed in detail below and in the Supplementary Discussion.

An initial expectation was that one or more of these genes would exhibit sexually-dimorphic dysregulation by VPA, thereby suggesting a plausible underlying mechanism for the sex difference in the incidence of ASDs; however, no significant sexually-dimorphic effects of VPA were observed.

Several genes affected by VPA in the fetal mouse brain appear in multiple GWAS studies (c.f., Tables 1 and 2) including *Adnp*, *Arid1b*, *Ash1l*, *Cdh2*, *Cdk5l*, *Dyrk1a*, *Kmt5b*, *Mecp2*, *Nr2f1*, *Pogz*, *Pten*, *Tbr1*, *Tcf7l2*, and *Wac*, moreover, nine of these genes were implicated in neurogenesis and cell fate determination in the embryonic brain ^76^. This “short list” is a potential starting point for future hypothesis-driven studies to determine whether dysregulation of one or more of these genes by VPA is a proximal cause of the behavioral abnormalities identified in the adult animals. At least 20 genes encoding components of intracellular signaling pathways are dysregulated by VPA in the E12.5 brain (Figure 2); disruption of these signaling pathways also has the potential to disrupt multiple developmental processes and may contribute to the autistic-like behavior induced by VPA. Considering that neurogenesis and the fate determination of excitatory and inhibitory neurons are occurring in the E12.5 mouse brain, genes involved in the regulation of these processes that are dysregulated by VPA (c.f., Table 3) are of particular interest. Nevertheless, it remains possible that dysregulation of one or more of the other genes altered by VPA contributes to the proximal cause of the autistic-like behavior. The role of these candidate genes in causing the autistic-like endophenotype could be tested by determining the effect of manipulating gene expression (singly or in combination) on VPA-induced behavioral abnormalities.

## Figures and Tables

**Table 1.** Autism-risk genes identified in five published studies ^42–46^. Genes dysregulated by VPA in fetal brain in the present study (Tables S2 and S3) are indicated in ***bold***. The percentage of VPA-regulated genes is shown for each study. 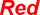: genes identified in all five published papers and **were** affected by VPA the present study; 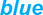: genes identified in four of the five published papers and **were** affected by VPA the present study; 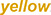: autism-associated genes identified in all five published papers but **were not** affected by VPA in the present study.

Satterstrom	Garcia-Forn	Yuen	Stessman	Ruzzo
***41%***	***50%***	***37%***	***41%***	***44%***
		*ADCY3*	*ADCY5*	
***ADNP***		***ADNP***	***ADNP***	***ADNP***
		*AFF2*		
		*AGAP2*		***AKAP9***
*ANK2*	*ANK2*	*ANK2*	*ANK2*	*ANK2* *
*ANKRD11*		*ANKRD11*	*ANKRD11*	
*AP2S1*				
***ARID1B***	***ARID1B***	***ARID1B***	***ARID1B***	***ARID1B***
***ASH1L***		***ASH1L***	***ASH1L***	***ASH1L***
***ASXL3***		***ASXL3***	***ASXL3***	
*BABRB3*				
***BCL11A***	***BCL11A***			***BCL11A***
*BRIN2B*				*BTRC*
*CACNA1E*				*C16orf13*
*CELF4*		*CACNA2D3*	*CACNA2D3*	*CACNA2D3*
			***CASK***	*CAPN12*
			***CDC42BPB***	*CCSER1*
***CHD2***		***CHD2***	***CHD2***	***CHD2***
*CHD8*	*CHD8*	*CHD8*	*CHD8*	*CHD8* *
		*CIC*		***CMPK2***
		*CLASP1*	*COL4A3BP*	
		*CNOT3*		
***CREBBP***				
*CTNNB1*	*CTNNB1*		*CTNNB1*	*CTTNBP2*
		*CUL3*	*CUL3*	*CUL3*
***DEAF1***	***DDX3X***	***DIP2C***	***DDX3X***	***DDX3X***
***DNMT3A***		***DNMT3A***	*DIP2A*	***DNMT3A***
*DPYSL2*			*DLG4*	
			*DLGAP1*	
*DSCAM*		*DSCAM*	***DOCK8***	*DSCAM*
		*DYNC1H1*	*DSCAM*	
***DYRK1A***	***DYRK1A***	***DYRK1A***	***DYRK1A***	***DYRK1A***
*EIF3G*	*FMR1*	*FAM47A*		***ERBIN***
				
				***FAM98C***
*FOXP1*	*FOXP1*	*FOXP1*	*FOXP1*	*FOXP1* *
***FOXP2***				*GABRB3*
*GFAP*				
*GIGYF1*		*GIGYF1*	*GIGYF2*	*GIGYF1*
*GNAI1*		*GRIN2B*	*GRIN2B*	*GRIA1*
*IRF2BPL*		*KDM6A*	*HIVEP3*	*GRIN2B*
***KCNQ3***			*ILF2*	*ILF2*
*KDM5B*			*ITPR1*	***INTS6***
***KDM6B***		***KDM6B***		***KDM6B***
*KIAA0232*		*KIAA2022*	*KATNAL2*	*KATNAL2*
		*KMT2A*	*KMT2A*	*KDM5B*
*KMT2C*		*KMT2C*	*KMT2C*	*KMT2C*
			***KMT2E***	***KMT2E***
***KMT5B***		***KMT5B***	***KMT5B***	***KMT5B***
***LDB1***			*LAMC3*	*MFRP*
***MAP1A***		***MECP2***	***MECP2***	*MLANA*
*MBD5*				
*MED13L*		*MED13*	*MED13L*	
*MKX*		***MYO5A***		***MYO5A***
*MYT1L*		*MYT1L*	*MYT1L*	*MYT1L*
*NRXN1*		*NAA15*	*NAA15*	*NCKAP1*
***NSD1***		*NLGN3*	*NCKAP1*	*NRXN1*
*PAX5*		*NLGN4X*	*NRXN1*	*P2RX5*
*PHF12*		*PAX5*	*PARD3B*	*PCM1*
*PHF21A*		*PCDH11X*	*PAX5*	
		***PHF2***	***PHF2***	***PHF2***
		*PHF3*	***PHIP***	
			***PLXNB1***	
***POGZ***	***POGZ***	***POGZ***	***POGZ***	***POGZ***
			*PPM1D*	*PRKAR1B*
***PPP2R5D***			***PPP2R5D***	
***PRR12***				
***PTEN***	***PTEN***	***PTEN***	***PTEN***	***PTEN***
***RAI1***	*SCN2A*	*SCN2A*	***PTK7***	***RANBP17***
*RFX3*			***PTPN11***	*RAPGEF4*
*RORB*			*RELN*	*SCN2A*
*SATB1*			*RIMS1*	
			*SCN2A*	
*SCN2A*			***SETBP1***	
			*SETD2*	
*SETD5*			*SETD5*	*SETD5*
***SHANK2***		***SHANK2***		***SHANK2***
*SHANK3*	*SHANK3*	*SHANK3*		*SHANK3*
***SIN3A***			*SLC6A1*	*SLC6A1*
*SKI*		*SLC6A1*	*SMC3*	*SMURF1*
*SLC6A1*				
***SMARCC2***		***SMARCC2***		
*SPAST*		*SPAST*	*SPAST*	*SPAST*
*SRPRA*				
		*SRSF11*	***SRCAP***	
			*SRGAP3*	
*STXBP1*			*STXBP1*	
*SYNGAP1*	*SYNGAP1*	*SYNGAP1*	*SYNGAP1*	*SYNGAP1* *
			*TANC2*	
***TBL1XR1***		***TAF6***	***TBL1XR1***	
***TBR1***	***TBR1***	***TBR1***	***TBR1***	***TBR1***
*TCF20*				
***TCF4***	***TCF4***		***TCF4***	
***TCF7L2***		***TCF7L2***	***TCF7L2***	***TCF7L2***
***TLK2***				***TMEM39B***
*TM9SF4*		***TNRC6B***	***TNRC6B***	***TNRC6B***
		*UBN2*	***TRIO***	
*TRIP12*			*TRIP12*	*TRIP12*
***VEZF1***		*UPF3B*	*UNC80*	***TSPAN4***
				*UIMC1*
				***USP45***
***WAC***		***WAC***	***WAC***	***WAC***
*ZYMND8*		***WDFY3***	***WDFY3***	***WDFY3***
			*WDR45*	*ZNF559*
			***ZC3H4***	
			*ZNF292*	

* Not affected by VPA

**Table 2.** Genes linked to autism in GWAS that have also been reported to regulate “neurite outgrowth”, “synapse/spine formation” or “synaptic plasticity” ^47^. Genes in red 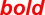 are also up- or down-regulated by VPA in the fetal brain (Table S3). Lin et al. ^47^ did not distinguish among the multiple known isoforms of cadherins (*CDH’s*); Cdh11 is downregulated 60% by VPA in the fetal brain (c.f., Table S3).

Neurites	Synapse	Plasticity
*CTTNBP2*	***ADNP***	***CDH(s)***
***CDH(s)***	***CDH(s)***	***CDKL5***
***CDKL5***	***CDKL5***	*DLGAP2*
*CNTN*	*CNTN*	*FMR1*
***CNTNAP2***	***CNTNAP2***	*GRIK2*
***DYRK1A***	*CTTNBP2*	***GRIK4***
*ELMO1*	*DLGAP2*	*GRIN2B*
*FMR1*	***DYRK1A***	***MECP2***
*GRIN2B*	*ELMO1*	*NLGN*
***MECP2***	*FMR1*	*NRXN*
*MYO16*	*GRIK2*	***PTEN***
*NCAM2*	***GRIK4***	*SHANK3*
*NLGN*	*GRIN2B*	***STXBP5***
*NRXN*	***MECP2***	*SYNGAP1*
*NTRK2*	*NLGN*	*UBE3A*
*PCDH*	*NRXN*	
***PTEN***	*NTRK2*	
*SPRICKLE1*	*PCDH*	
***STXBP5***	***PTEN***	
*TSC1*	*SHANK3*	
***TSC2***	*SYNGAP1*	
*UBE3A*	*TSC1*	
	***TSC2***	
	*UBE3A*	

**Table 3.** Biological roles of 189 different genes that are dysregulated by VPA in the fetal brain and associated with autism or prenatal neurodevelopmental processes (from Tables S3 and S4). Note that some genes are included in more than one category. The number in parentheses shows the FC induced by VPA (mean of males and females); 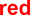 text indicates that VPA increased gene expression The basis for this categorization, with references, is given below and in Supplemental Discussion.

Intracellular signaling	*Ak2* (+1.3), *Akap8* (−1.3), *Akap8l* (+1.6), *Akt1* (−1.4), *Akt2* (−2.1), *Col6a6* (−4.1), *Dyrk1a* (−1.5), *Dyrk1b* (−2.2), *Grb2* (+1.4), *Kras* (+1.4), *Map2k1* (+1.7), *Mapk1* (+1.3), *mTor* (+1.4), *Ndst3* (−1.6), *Pik3cb* (+1.8), *Pik3cg* (−2.3), *Pik3r4* (+1.3), *Plcg2* (+1.8), *Pten* (+1.4), *Raf1* (−1.6), *Rmnd5b* (+1.3), *Rnf19b* (+1.4), *Sos1* (+1.3), *Stat3* (+2.7), *Tsc2* (−1.3)
Neurogenesis	*Adnp* (−1.6)*, Arid1b* (−2.2)*, Ash1l* (−1.6), *Asxl3* (−1.7), *Baz2b* (−1.5), *Bcl11a* (−1.6), *Chd2* (+1.3), *Cdk5rap2* (−1.3), *Ddx3x* (−1.6)*, Ebf1* (−1.4), *Eomes* (*Tbr2*) (−1.8)*, Fgf17* (−1.6), *Foxc1* (−1.9), *Hnrnpk* (−1.3), *Hnrnph1* (−1.6), *Hnrnpu* (−1.6), *Irf2bpl* (−1.4), *Kat2b* (−1.7), *Kdm6b* (−1.4), *Kmt5b* (−6.5), *Mecp2* (−1.6), *Lrrk1 (−2.9), Neurog1* (−2.2), *Plk1* (−1.7), *Pogz* (−2.2), *Smarcc2* (−1.6), *Srcap* (−3.5), *Tbl1xr1* (−1.5), *Tbr1* (−2.8), *Trnp1* (+1.5), *Wac* (−1.4), *Wnt3a* (−3.8), *Zbtb16* (+1.8)
Excitation-Inhibition	*Arid1b* (−2.1), *Camk2a* (+1.9), *Cc2d1a* (−1.4), *Csde1 (−2.0), Gad1* (−2.5), *Gad2* (−3.3), *Gabra1* (−1.7), *Gabra2* (−1.7), *Gabra4* (+2.5), *Grin2a* (−2.3), *Insyn1* (−1.4), *Kmt5b* (−6.5), *Maf* (−1.6), *Mafb* (−1.6), *Nf1* (−1.3), *Nr2f1* (*Coup-tf1*), (−1.8)*, Plxnb1* (−1.8), *Sema4a* (−1.6), *Sema4d* (+1.9), *Sfrp2* (−1.8), *Slc17a6* (−2.4), *Slc17a7* (+1.6), *Slc38a1* (−1.7), *Trpm4* (+2.1)
Calcium signaling	*Calm1* (+1.4), *Cacna1b* (+1.4), *Cacna1c* (−1.4), *Cacna2d1* (−1.7), *Cacnb2* (−1.9), *Camk1* (−1.6), *Camk2a* (+1.9), *Camkk1* (+2.6), *Camkv* (+2.4)*, Ppp3cb* (−1.3), *PPP3cc* (−1.3)
Retinoic acid signaling	*Megf10* (−1.9), *Cntnap2*, (+1.3), *Meis2* (−1.4), *Cbln2* (−1.8)*, Rxrb* (−1.3), *Rarb* (−2.2)
Neuronal fate specification	*Ebf3* (−1.6), *Neurod1* (−1.5), *Nkx2–2* (−1.8), *Nr2f1* (*Coup-tf1*), (−1.8), *Olig1* (+2.1), *Tbr1* (−2.8)
Axon growth/guidance	*Draxin* (−1.5), *Hnrnpab* (−1.6), *Marcks* (−2.2)*, Slitrk1* (−1.5), *Syn2* (+1.7)
Neuronal migration	*Apc2* (−1.7), *Mllt11* (−1.5), *Neurod1* (−1.5), *Nsd1* (−1.4), *Otp* (−2.5), *Ptk2b* (−1.7), *Robo4* (−2.2)
Synaptogenesis	*Arc* (+1.6)*, Cbln2* (−1.8)*, Csde1* (−2.0), *Ddx3x* (−1.6), *Hapln1* (−3.9), *Hapln4* (+25.7), *Pogz* (−2.2), *Slc17a6* (−2.4), *Slc17a7* (+1.6)*, Slitrk5* (−1.6), *Slitrk4* (+1.7), *Syncrip* (−1.4), *Top3b* (−1.5)
Dendrite development	*Cc2d1a* (−1.4), *Csde1* (−2.0), *Disc1* (−1.9)*, Dlg5* (−3.0)*, Gas7* (−1.7), *Mef2d* (+1.5), *P2rx7* (−2.0)*,* *Sema3a* (−2.0), *Slitrk1* (−1.5)*, Tmem163* (−1.7), *Twf2* (+1.6), *Zbtb16* (+1.8)
Postsynaptic cytoskeleton	*Homer1* (+1.6), *Homer3* (−1.4), *Shank2* (−2.3)
Dopaminergic system	*Adora2a* (−1.3), *Bdnf (+2.8), C1qtnf1 (−1.4), Csf1r* (−1.4), *Drd2* (+2.0), *Emp2* (−1.6), *Foxa1* (−1.6), *Klf4* (−1.6), *Lmx1a* (−1.7), *Mcam* (+1.7), *Palm3* (+1.9), *Ppp1r1b* (+3.7), *Robo4* (−2.2), *Rtn4rl1 (+2.0), S1pr1* (−1.3), *Sfrp2* (−1.8), *Slc9a3r1* (+3.0), *Smyd2* (−1.3), *Sparc* (−1.4), *Srebf1* (+1.4), *Th* (−1.4), *Tmem108* (+1.4), *Wnt5a* (−2.7), *Wnt7b* (−1.8)
Cholinergic system	*Chrna7* (+1.4), *Gbx1* (−2.3), *Isl1* (−1.5), *Lhx8* (−2.7), *Nkx2–1* (−2.5),
Endocannabinoid system	*Cnr1* (−2.0)
Autism and Down syn- drome	*Brwd1* (−1.3), *Cbs* (+5.0), *Dop1b* (+1.4), *Dyrk1a* (−1.5), *Erg* (−2.4), *Ets2* (−1.4), *Pigp* (−1.6), *Setd4* (−1.9), *Shebgr* (+1.8), *Sim2* (−2.2), *Wdr4* (−1.4)
Circadian rhythms	*Arntl* (+2.1), *Cry1* (+2.5), *Cry2* (+2.0), *Fbxl21* (+3.2), *Fbxl3* (−1.8), *Gabra4* (+2.5), *Gad1* (−2.5), *Gabbr2* (−2.4), *Mef2d* (+1.5), *Per1* (+1.9), *Per2* (+1.7)
Neuroinflammation	*Cd200* (−2.0), *Dnajc12* (+4.5), *Hspa1a* (+5.9), *Hspa1b* (+5.6), *Hspa2* (+6.2), *Igf1* (−3.2), *Igf2* (−2.7), *Tgfb2* (−2.4), *Tgfb3* (−2.0), *Tgfbr2* (−2.5)
Epigenetic regulation	*Adnp* (−1.6)*, Arid1b* (−2.2)*, Ash1l* (−1.5), *Bcl11a* (−1.6), *Brd1* (−1.4), *Brd2* (−1.3), *Ehmt1* (−1.4), *Kat2b* (−1.7), *Kmt5b* (−6.5)*, Kdm6b* (−1.4), *Mecp2* (−1.6), *Setd1b* (−2.2), *Smarcb1* (−1.7), *Smarcc2* (−1.6)
